# Conceptual and terminological confusion around personalised medicine: a coping strategy

**DOI:** 10.1186/s12910-016-0122-4

**Published:** 2016-07-18

**Authors:** Giovanni De Grandis, Vidar Halgunset

**Affiliations:** Department of Philosophy and Religious Studies, Norwegian University of Science and Technology, NTNU Dragvoll, 7491 Trondheim, Norway

**Keywords:** Personalised medicine, Definition, Conceptual confusion, Research policy, Healthcare policy, The politics of naming, Systematic literature review, Methodology, Medical ethics, Contextual meaning

## Abstract

**Background:**

The idea of personalised medicine (PM) has gathered momentum recently, attracting funding and generating hopes as well as scepticism. As PM gives rise to differing interpretations, there have been several attempts to clarify the concept. In an influential paper published in this journal, Schleidgen and colleagues have proposed a precise and narrow definition of PM on the basis of a systematic literature review. Given that their conclusion is at odds with those of other recent attempts to understand PM, we consider whether their systematic review gives them an edge over competing interpretations.

**Discussion:**

We have found some methodological weaknesses and questionable assumptions in Schleidgen and colleagues’ attempt to provide a more specific definition of PM. Our perplexities concern the lack of criteria for assessing the epistemic strength of the definitions that they consider, as well as the logical principles used to extract a more precise definition, the narrowness of the pool from which they have drawn their empirical data, and finally their overlooking the fact that definitions depend on the context of use. We are also worried that their ethical assumption that only patients’ interests are legitimate is too simplistic and drives all other stakeholders’ interests—including those that are justifiable—underground, thus compromising any hope of a transparent and fair negotiation among a plurality of actors and interests.

**Conclusion:**

As an alternative to the shortcomings of attempting a semantic disciplining of the concept we propose a pragmatic approach. Rather than considering PM to be a scientific concept in need of precise demarcation, we look at it as an open and negotiable concept used in a variety of contexts including at the level of orienting research goals and policy objectives. We believe that since PM is still more an ideal than an achieved reality, a plurality of visions is to be expected and we need to pay attention to the people, reasons and interests behind these alternative conceptions. In other words, the logic and politics of PM cannot be disentangled and disagreements need to be tackled addressing the normative and strategic conflicts behind them.

## Background

Since the Human Genome Project the prospect of using genomics (as well as other ’omics) to make medicine more precisely targeted to the particular biology of individuals and their diseases has fuelled hopes, ambitious initiatives and mobilised considerable funding.^1^ While several different labels have been used—like precision medicine and P4 medicine—one has proved especially popular and successful: **Personalised Medicine** (henceforth PM). As many have pointed out [1–9], doctors have always practiced with the aspiration to respond to individual variability and to tailor care and treatment to the specific needs of each patient. Nevertheless the popularity of the concept in biomedical sciences, health policy, science policy and in public discourse is a recent phenomenon that has been largely stimulated by advances in genomics and molecular biology [10–13].^2^ As a concept that concerns a broad range of stakeholders—scientists and policymakers, healthcare workers, the pharmaceutical and diagnostics industries, IT network providers and users, health insurances and taxpayers, not to mention patients themselves—it is subject to a variety of different uses and interpretations [14, 15]. With this kind of variety typically comes the Babel worry: are people speaking the same language and understanding each other? This worry has generated a number of attempts to analyse the meaning of PM, either with the aim of facilitating better communication and understanding or in the attempt to promote or criticise particular uses [9, 12, 13, 16–20].

So far the most influential analysis has been the paper by Sebastian Schleidgen and colleagues [12] who have attempted to accurately specify the nature and boundaries of PM.^3^ They pursue this goal through a systematic literature review (henceforth SLR), aiming to extract from this a more precise definition of PM. No doubt their attempt addresses a genuine need for some guidance through a debate that is not easy to approach. The sheer number of publications, as their literature survey attests, is daunting; the field is still young and rapidly evolving; but most importantly there is lack of consistency in the use of terminology and concepts. As noted above, PM has also been named *precision medicine*, *stratified medicine*, *genomic medicine*, *personalised molecular medicine*, *individualised medicine*, *P4 medicine*, *personalised healthcare*—and this is not even an exhaustive list. The scope of the concept is equally variable, and sometimes explicitly disputed: some use it to refer only to the new possibilities offered by molecular biology to improve diagnosis and therapy on the basis of individual biological differences, some use it much more comprehensively to envision a new paradigm of healthcare.

Against this confusing backdrop Schleidgen and colleagues’ effort has considerable appeal, insofar as a systematic literature review promises a solid evidence base for clarifying the issue. Their study moreover claims to have important practical implications: according to the authors their definition provides “an adequate basis for public discourse on PM” ([12]: p. 2) thus facilitating legislators in drafting regulatory mechanisms and making policy decisions, preventing the public from developing misplaced hopes and fears, and last but not least preventing stakeholders from pursuing their own interests above patients’ needs. Another important achievement that they claim for their work is to have established the proper scope of the concept: “PM can only be understood as an add-on to standard medical care” ([12]: p. 10) and “is *not* medicine with a special focus on the interests and preferences of the individual patient. … Hence PM as such is not related to the term patient-centred medicine” ([12]: p. 11). The definition at the basis of these claims is the following:

*PM seeks to improve stratification and timing of health care by utilizing biological information and biomarkers on the level of molecular disease pathways, genetics, proteomics as well as metabolomics* ([12]: p. 10).

Their emphasis is thus fully on the side of the development of new scientific and technological means that will merely enrich the toolkit of medicine.^4^ This contrasts with a trend within the PM literature towards a broader understanding of PM, namely an understanding that while centred on the advances of molecular biology, includes within its remit its clinical translation, implementation, integration within the healthcare system and its funding [1, 3, 21–27]. Similarly, their resolute disconnection between PM and patient-centred care does not go unchallenged in the literature. Some authors have put forward plausible reasons for integrating PM and (some features of) patient-centred care [9, 28–35]. Others have questioned the use of the label “personalised medicine” only for *biologically* personalised medicine, arguing that the concept needs to have a broader and non-reductionist understanding of “the person”—along the lines advocated by patient-centred care [8, 19, 34, 36–39]. In light of these opposing views, we think that Schleidgen and colleagues’ bold and ambitious attempt to provide such a strict definition deserves close scrutiny. Is their case robustly grounded and has their method really given them an edge over other writers arguing for opposite conclusions? We conclude that it has not. Our analysis indicates that disagreements and conflicts around the scope and meaning of PM cannot be solved by producing a better definition: for there is more than logic and semantic at stake. Hence we urge to map and understand the theoretical and practical (i.e. ethical, economic, political, professional) reasons and roots of disagreements. In this way, if we are lucky, we may promote mutual understanding and reasonable accommodations, and if the goings get though we may expose some of the cunning of power and vested interests.

## Discussion

### Outline of the argument

Since our argument is rather long and structured in several steps, readers may find it helpful to have the plan of the discussion clearly laid out at the outset. We begin by questioning Schleidgen and colleagues’ use of a SLR in the “methodological critique” section, which is articulated in three stages. We first question the epistemic robustness of definitions and the lack of selection criteria that pay attention to how definitions are produced and which purpose they serve. Then we examine the process of extracting a more precise definition and argue that the logical principles used to filter out noise are not normatively neutral and beg some questions. We further argue that Schleidgen and colleagues’ evidence base is too narrow, for they have confined their review to only one type of literature to the effect that their results are partial towards the point of view of one group of stakeholders. The conclusion of this long initial section is that Schleidgen and colleagues’ use of a SLR has some rhetorical force, but actually does not provide robust and compelling grounds for a normative definition.

In the next section, “philosophical critique”, we contend that they have misplaced hopes in the power of definitions—because having a solution accepted in the real world is not simply a matter of offering philosophical arguments—and a mistaken belief that concepts can be defined without paying proper attention to the context of use and the purposes of users. In the following section we make a more general point and call attention to the distinction between two related but different disputes about PM: namely the question of the extension of the concept (and drawing the boundaries with neighbouring ones) and the question of what is the most appropriate name for what we refer to as PM. These are not merely “logical” questions: they are ethical and political as well since they affect the roles and responsibilities of different stakeholders and hence their power and leeway.

Our argument culminates in the “values and pluralism” section, where in an almost Hegelian spirit we attempt to show that once we appreciate the reasons why “PM” is such a messy concept and why people wrestle around it and put forward competing visions, then we may make sense of the apparent confusion, see the rationale behind it and the costs of getting rid of it.

Any attempt at disciplining the use of a contested concept is unlikely to be neutral in its implications or acceptable to all stakeholders. Because of their attempt to pin down and restrict the concept, Schleidgen and colleagues fail to give all voices a fair hearing and unwittingly favour those of some stakeholders. In the attempt to stay clear of stakeholders’ interests for the sake of neutrality they have made themselves blind to the normative and non-neutral implications of their own analysis. We agree that philosophers should aim to lessen confusion—though not by ignoring complexity or prematurely adjudicating between contenders—but by making this complexity and the alternative perspectives that constitute it more intelligible.^5^ Therefore, we propose to see PM as a work in progress and the label for a cluster of visions for the future of healthcare (cf. [14, 18, 40]). One of its key functions today is to bring together stakeholders and in order to do that it has to remain open to different and sometimes conflicting interpretations [16, 41]. This openness itself, though not without its dangers, is valuable in that it may help to prevent that the preferences and aims of only a subset of stakeholders shape the language and the agenda giving them a hegemonic position. Once we acknowledge the contestedness of PM, decisions that adjudicate between conflicting interests have to be justified through fully engaging in normative debates. The clarity and fairness of such normative work can be greatly enhanced by a preliminary philosophical mapping of the values and interests that motivate different actors.

### Methodological critique

We believe that the high impact of Schleidgen and colleagues’ paper is largely due to their having based their conclusions on a systematic literature review, a method that over the last three decades has achieved a very high status. As is well known, together with randomised controlled trials (RCTs) and meta-analyses, SLRs are the primary tools of Evidence Based Medicine and of Evidence Based Policy, two prominent trends in the medical and policy domains respectively. The importance of SLR is well exemplified by the key role they play in the work of the Cochrane Collaboration and of the Campbell Collaboration: the two most authoritative and emblematic institutions in supporting evidence-based medicine and evidence-based social policy and in disseminating their results [42, 43]. SLRs have earned their reputation as powerful tools for providing reliable evidence and solid empirical grounding, especially when performed on literature reporting the results of RCTs. These latter provide the solid experimental evidence while SLRs conveniently synthesise their results. The robustness of their results depends on the quality of the studies reviewed: SLRs are subject to the principle *garbage in, garbage out*. This is why SLRs often need to mediate and make trade-offs between two desiderata: synthesising *all* the evidence available vs. synthesising the *best* evidence available [44, 45]. The former avoids introducing any bias, the latter avoids relying on poor quality evidence. When the latter option is chosen the criteria of exclusion become very important and have to respect widely acknowledged principles of methodological robustness.

#### Epistemic robustness

It is important to keep in mind why and under which conditions SLRs deliver robust results. This is achieved when the set of reviewed studies includes either a large number of methodologically sound small RCTs or a smaller number of methodologically sound large RCTs.^6^ We will call reviews that meet these criteria epistemically robust SLRs. To what extent does the review conducted by Schleidgen and colleagues meets the conditions that secure the robustness of its results?

SLRs typically analyse reports of experimental results or other empirical inquiries. In dealing with their sources assessing and weighing their strength is paramount. This is done along two dimensions. Reviewed studies are assessed on the breadth of their evidence base and the robustness of their method. The larger the evidence base and the more robust the method, the more weight is accorded to a study. A small observational study cannot count as much as a large RCT. Schleidgen and colleagues do not analyse reports of experimental results, but academic papers featuring a definition of PM. This makes their SLR atypical. What is more questionable, they do not apply any selection or classification criteria to their material. They simply aggregate PM definitions as if they were all arrived at through rational procedures as reliable as an RCT. Our objection is that, just as different empirical studies must be assessed differently and ranked in order of epistemic merits, so too must definitions be approached with sensitivity to how they were arrived at. We should be equally sensitive to the fact that the purpose a definition serves is likely to vary. The following list of examples is not meant as an exhaustive taxonomy, it simply shows that even in the academic debate definitions constitute a heterogeneous category of variable quality and epistemic strength.^7^

Sometimes PM definitions are used by authors to specify their own use of the term in a given paper (e.g. [46, 47]). Assuming that the authors manage to use it consistently, a definition of this kind is robust; however its “evidence base” is limited since it can claim only to apply in *one* case. Then there are papers where the authors define PM in order to characterise how this phenomenon is commonly understood (e.g. [3, 48]). Often these are expert opinions, offered by scholars who can claim familiarity with the field and acknowledged expertise. However, even if these definitions are often supported by references to the literature, they are not necessarily worked out according to systematically collected evidence. This marks them off as “less robust” than definitions of a third kind. Some PM definitions that aim at describing actual use boast both a broad evidence base and derivation from well-established methods, e.g. comprehensive literature reviews. The paper of Schleidgen and colleagues fits in this latter category (cf. also [13, 27]). Yet their methodology passes over such epistemic differences and overlooks that definitions vary in nature and quality. Treating all definitions as equals leads to the striking and paradoxical result that in an iteration of their systematic review their own definition (based on reviewing more than 1400 definitions) would count no more than a definition describing a rather idiosyncratic use or one casually dropped in an editorial. This epistemic blindness seems to us a betrayal of a key methodological rule of SLRs.

#### Logical issues

Another departure from the conditions of epistemic robustness is that Schleidgen and colleagues do not simply synthesise the findings: they want to come up with a “precising” definition, i.e. they want to single out PM’s core meaning so as to reduce vagueness. In order to compensate for the lack of quality criteria for inclusion and to produce an improved definition, Schleidgen and colleagues have to combine the extraction of results with a process of “noise removal”. The processing of their inputs (the definitions found) happens in two stages. The first is an extraction of features organised along the categories of ends and means of PM, the second is a filtering through the application of “logical” criteria. Roughly speaking we have first an analytical dissection (*resolutio*) of definitions, and then a synthetic recombination (*compositio*) guided by some normative principles. We are going to focus on the recombination process, because it is the most unusual and original feature of their SLR, as well as being the “logical laundromat” that turns inputs of variable quality into an output of higher quality and value.

The authors employ “six criteria of adequate definitions” ([12]: p. 5) that are then used to filter the definitional features found in the literature. Focusing on the first two principles will be enough to make our point. The first is that “a definition must be necessary, i.e. there must not exist any well-established term equivalent with its *definiens*” ([12]: p. 5). This principle raises a question that the authors completely ignore. As mentioned, several other names have been used to indicate what they define as PM. Considering that there was a different use of PM in the medical literature (see [49–51], cf. [52, 53]) and that patients often attribute to it a meaning different from those prevailing among medical researchers ([34, 39, 54: pp. xiii-xiv, 55: p. xii]), it is not clear why they do not even consider adopting one of these synonyms rather than the ambiguous PM. After all it seems a fair corollary of the principle of necessity that one should not use a name already in use if equally valid alternatives are available and thereby avoid unnecessary multiplications and confusions. Adopting instead, say, the label “stratified medicine” would avoid misunderstandings and associations with person (or patient)-centred medicine (that seem particularly invidious to Schleidgen and colleagues) and also prevent unfounded hopes of genuinely individualised ’omics therapies [56]. More generally, their principle of necessity seems to us to call for a serious discussion of the fact that their definition (i.e. their *definiens*) could be used for *stratified medicine* and *precision medicine*. We find surprising that Schleidgen and colleagues neither tackle this problem nor attempt to consider what is the relation between PM and related concepts. This is not a criticism of the principle itself, but of the fact that Schleidgen and colleagues fail to apply it thoroughly. Taking the principle seriously should raise the question not only of defining the concept, but also of how to name it, since many different names have been used for the concept here referred to as PM.

That said, the criterion of necessity is not unproblematic in the context of the task the authors set for themselves. By requiring that what is described by the definiens is new, the criterion rules out the possibility that PM has always been there and is nothing new. This was one of the competing understandings of PM mentioned at the beginning of their paper and it seems illegitimate to simply rule out one of the contending interpretations through some logical stipulation. Would it be logically incorrect to conclude that PM is something broader and more generic than what they have defined, while what they have defined is, say, ’omics medicine? If not (and the authors provide no reason for thinking that it is) then it should not be ruled out by some criterion for adequate definition. It is peculiar to want to determine whether PM is something new or not and then to assume that it *has* to be something new.^8^

The second logical criterion they propose is that “a definition must be neither too broad nor too narrow” ([8]: p. 5). This evidently leaves a lot of scope for the judgment of the authors and hence introduces a strong subjective element. For instance they make some choices on the basis of their understanding of what falls within and outside the boundaries of medicine, and their interpretation is clearly open to question, as when they claim that “prevention and therapy are what constitute health care” ([8]: p. 10), a position that has the rather controversial implication of excluding palliative care from health care.^9^

#### Selection bias

Perhaps the most important reason why their SLR is not up to the task set by the authors is its narrow scope. The authors sensibly want to ground their definition on the actual use of the term, but then questionably limit the survey to “[PM] definitions appearing in the academic literature” and to “its current use in the sciences” ([12]: p. 2). They themselves acknowledge that PM “has become a buzz word in the academic as well as public debate surrounding health care” ([12]: p. 1). Hence actual use is clearly not confined to the academic literature. Schleidgen and colleagues justify excluding “the stakeholders’ discourse” from their survey by wanting to exclude vested interests and ill-founded hopes. What they want is to focus on the “actual scientific possibilities as well as limitations of medical measures labeled as PM” ([12]: p. 2). We are not convinced by this argument. One reason of perplexity is that if they want to avoid hype they should not only exclude what is beyond the scientific limits, but also what is non viable because it is financially not sustainable, ethically unacceptable for patients or healthcare providers, incompatible with deeply entrenched professional cultures and practices etc. (cf. [57, 58]). Another and more serious problem with their argument is its naive assumption that biomedical literature is immune from stakeholders’ interests. It is hard not to see that the research community has a great stake in PM and that they are as vulnerable to the temptation of hype as any other stakeholder, to the point that social scientific and historical looks at PM have emphasised the key role of expectations in gaining momentum and shaping actual developments [11, 14, 15, 40, 58–62]. For fear of stakeholders’ vested interests they have appointed one group of stakeholders as the only authoritative voice, thus leaving their interests uncontrolled by any check and not counterbalanced by conflicting interests.^10^

Taking a more inclusive perspective is possible, since others have looked at different stakeholders groups and captured different voices. Lada Leyens and colleagues [27] have shown that a literature review can be done of policy documents about PM—and the results are rather different from those of Schleidgen and colleagues. McFarland and colleagues [39] and Heusser [34]—drawing from a Swiss study—provide some empirical evidence of patients’ views of PM, and a survey of General Practitioners’ views would be necessary to test how popular is the view that PM “has always been a component of good medical practice”, “based on the relationship between patient and physician rather than on any particular technology” ([37]: p. 1684). Views vary not only across stakeholders but also across circumstances, to the effect that the same stakeholders may express differing views according to their interlocutors. For instance, Susanne Michl [14] has shown that academic authors provide different visions of PM when they write for their peers and when they address the lay public. The list of stakeholders could be made longer, but the essential point is clear: any definition that reflects only a subset of uses (e.g. the internal discourse of biomedical researchers) is bound to be biased.

Instead of producing a PM definition robustly grounded in empirical evidence, Schleidgen and colleagues have ended up making methodologically dubious moves and producing a definition grounded in only one source of evidence. It is revealing that at the end of their paper Schleidgen and colleagues seem to recognise some of these shortcomings. They admit that PM cannot be treated simply as a scientific concept, because it has already established itself outside the purely scientific discourse. When scientists step out of their laboratories, “Personalised Medicine” will inevitably evoke (unpredictable) associations and expectations. Surprisingly, they therefore conclude by proposing to abandon the term altogether (“To forestall false hopes attached to the concept and accordingly wrong decisions regarding investments, it might be reasonable to adapt terminology” ([12]: p. 12)) and to adopt “stratifying medicine” instead—and indeed we agree that stratified medicine is the more accurate label. Nevertheless we conclude that a discourse analysis might have been a more appropriate method for achieving their goal and that carrying out a SLR has not in itself made their analysis and proposal more compelling than alternative ones.

### Philosophical critique: concepts and meaning

Having noted problems with the authors’ choice of method and the result (the definition) it yields, in this section we move on to the further question whether a definition could possibly have achieved the result they were aiming for. We argue that their project is hampered by philosophically dubious ideas about definitions and the workings of language more generally, and also that it reflects a lofty view of the importance of conceptual analysis.

The authors start by describing PM as a hopelessly vague term, and aim therefore to help “structuring the debate over PM’s meaning by developing a *sufficiently* precise definition” ([12]: p. 2). Sharper concepts may be helpful in giving a more coherent structure to a diffuse debate. However, in practice the quality of one’s analysis is not the main factor determining what influence one has on a debate. What impact (if any) their definition will have on the subsequent PM debate ultimately depends on how it is received by the pertinent readers. Unless this more focused definition is also *seen* as suitably focused (and thus as an improvement) by a sufficient number of stakeholders, its effect on the debate is likely to be limited. Confident in their undertaking, the authors anticipate some disagreements—but only minor ones, since, according to them, the majority already understands PM more or less in line with their definition ([12]: p. 11).

The suggestion is that their definition may indeed succeed in structuring the subsequent debate because the debate *already* is (largely) structured around the stratification of treatment on the basis of molecular biology. This is puzzling. By claiming that their definition ought to be acceptable to the majority since it converges with the dominant understanding,^11^ the argument seems to invite two interpretations: either their solution works only under the assumption that the problem is already solved—which is no solution at all—or there is no major disagreement to speak of in the debate—which is to say that there never was a problem to be solved in the first place. The authors may accept the latter interpretation, not as an objection but rather as an account of what they have discovered, namely that at the centre of the debate, obscured by inconsistent terminology, there is major agreement on PM’s meaning. Hence what their definition actually does is to bring this common ground into sharper focus. This move would be untenable, however, because their empirical evidence does not warrant such a conclusion. In the previous section we observed how the authors selectively reviewed only academic papers; but what prompted their hunt for a sharper definition was the cacophonic impression created by various stakeholders using PM to signify different things in “the academic as well as public debate surrounding health care” ([12]: p. 1)—and they have not provided any reason for believing that the majority in this broader context will find their results acceptable. Consider this conclusion:

As our results show, PM is *not* medicine with a special focus on the interests and preferences of the individual patient. For instance, PM does not include any reference to an adequate doctor-patient relationship. Hence, PM as such is not related to the term patient-centered medicine. ([12]: p. 11)

If this is meant to be a *statistical* remark about the use in *scientific* literature, it is correct. We are not contesting their empirical findings, merely emphasising the obvious point that what they found is a function of where they chose to look—as other studies suggest [34, 39, 63] a survey among patients or family doctors would probably have yielded different results. What they can reasonably claim to have shown is that references to adequate doctor-patient relationships are uncommon in the scientific literature about PM, and that, in the context of scientific reporting on new findings in genomic and molecular biomarkers, PM can be defined in purely molecular terms. But the more ambitious claims about this being the “true” meaning of PM cannot be supported, since if PM is a notion used in different contexts and by different actors, then what PM is and should be cannot be determined by extrapolating from the use of one group of stakeholders in one specific context.

At the roots of this mistake is a philosophical assumption about language: that words may be sharp or vague in themselves. The paper is subtitled “sharpening a vague term”, and the suggestion is that the lack of a precise definition is responsible for the varying uses of “PM”. Lack of a fixed meaning, however, is not peculiar to PM. Consider the concept “game”: there are board-games, card-games, ball-games, computer games, Olympic Games and children’s games; we may speak of mind games, game shows, the game of politics; philosophers discuss language-games [64] and psychologists sometimes talk about social games (as in “the games people play”, [65]). A continuum of uses may of course make a concept philosophically confusing and difficult to pin down; but this does not necessarily mean that either the term or people using it are confused. Miscommunication can of course arise when people using the same concepts differently come together. Tempting as it may be, philosophical abstraction is not the solution, because the meaning of words is generally determined by where and why words are used. When is a knife sharp enough? It depends on the context. Depending on the work it is meant to do, the same knife may be both dull and sharp—in the hands of a child practicing wood carving it may be dangerously sharp, while it may still be hopelessly dull for proper fish filleting. Similarly, it is futile to attempt a precise definition of “PM” without a fairly definite context in mind, since distinguishing between precise and imprecise uses of a word is possible only when the aims and purposes are clear. Misunderstandings and disagreements will have to be solved when they arise by people patiently negotiating their meanings and purposes. There is no general philosophical quick-fix (for a philosophical discussion see [66–68]).

Therefore it makes no sense to stipulate the rules for how “PM” should be used without simultaneously describing the circumstances under which those rules would apply. Schleidgen and colleagues’ definition is in this respect an abstraction—both in the pejorative sense of being other-worldly and in the etymological sense of being disconnected from its proper surrounding. Their aim is to provide “an adequate basis for public discourse on PM”, but unfortunately they leave the notion of “public discourse” too vague and unspecified to provide a meaningful context: sometimes public discourse is presented in contrast to academic debate, sometimes it seems to include this latter. We are told nothing specific about this public debate: what it is about, what is at stake in it, who the stakeholders are, what their converging and conflicting interests are? All these crucial questions are left unanswered. As a result what could count as a sufficiently precise definition in such a hazy context is very hard to figure out. Taking seriously the variety of actual uses, one should not assume that a concept like PM *must* be held together by some common core, for the simple reason that in so far as this is partly an empirical question one must remain open to the possibility that there may be no *one* thing in common to *all* uses of “PM”.^12^ Instead of trying to reduce the complexity by determining rules for the use of “PM”—as if one rule could be valid universally or prohibit the word from travelling and thus taking on new meanings—a more sensible way to try to clear the fog would be to contextualise the various uses so that we can achieve a clearer view of the aim and functioning of the words.

### Two sources of disagreement

Once discussions are viewed more in context it is easier to appreciate that there are two kinds of disputes around the notion of PM. The first concerns the extension or scope of the concept: i.e. discussions about what it includes and what lies outside its remit. These conceptual disputes are often tackled by producing a taxonomy of concepts and placing PM within it (see for instance [13, 17, 19]). The other dispute—often arising in reaction to a narrow understanding of PM as genomic medicine—centres on whether “PM” is the appropriate label or rather a misnomer or an objectionable expropriation of a name with a broader meaning. Some authors resent the fact that the words “personal” and “personalise” that used to belong to clinical practice—especially to primary care—have been appropriated by biomedical science, which is thus exploiting its rhetorical force and emptying it of its critical potential to oppose narrow and reductionist approaches to medicine [19, 34, 38].

One issue is how to organise the logical space, i.e. what kind of conceptual grid is needed in order to specify most effectively the boundaries and relations between neighbouring concepts, e.g. to establish whether PM and Genomic Medicine are co-extensional, mutually exclusive, partially overlapping or one subsuming the other. For instance, Pokorska-Bocci and colleagues [13] suggest a grid with 4 basic concepts subsumed by one more general concept, which in turn is subsumed under another still more general one. But once the grid has organised the relations between concepts, there is the question of what is the most appropriate name for each cell of the grid (i.e. for each concept). For instance, if a distinction has been drawn between attempts at personalising medicine through 'omics and through other means the question arises whether either of the two should be called “personalised medicine”—or whether PM should be used as a broader concept encompassing both. Reasons for and against each possibility can be given. Calling ’omics medicine “PM” has the advantage of reflecting a common use, but the disadvantage of not being very accurate because arguably what can be achieved is stratification of patients rather than personalisation [69, 70, 71]. Using PM only for personalising measures not based on molecular biology goes against what is now a widespread use, but it could be a way of reclaiming a name that has been captured by molecular medicine [19, 38]. Using PM as a broader category may seem a fair and conciliatory choice [13], but again it may be confusing given the prevailing current use—that’s why “personalised healthcare” is often preferred for this purpose [9, 17]. As it already emerges from these examples, questions of naming are affected by a diverse range of considerations: semantic clarity, consistency with established uses, but also grievances towards the practices with which they are connected, and considerations of fairness and impartiality. Lexical choices have as much to do with ethics and politics as they have with logic and semantics. Furthermore how these multiple criteria are balanced and weighed is open to reasonable disagreement ([72]: II, §2).

Another source of indeterminacy lies in the fact that *PM is very much a future-oriented concept*. More than an existing reality, PM is currently the controversial label of a cluster of visions for (some aspects of) the future of healthcare. Future-oriented concepts, aimed at mobilising initiatives and building networks, are bound to be fluid and contested. Given that it is still something in the making, what PM will become depends on what the technologies can actually deliver and which decisions are made, which in turn depends on who gets involved, which ideals, values and interests different stakeholders want to realise, which alliances are formed among stakeholders, which compromises are made, which scientific discoveries take place etc. [14, 18, 59]. In view of this, the attempt to decide what PM will become is not only premature, but also fails to understand the current grip of the term. By trying to structure the debate around a predefined and limited range of topics one obscures the fact that which people are using the concept and the contexts in which they do so are key determinants of its meaning. This is why *attention should be refocused towards stakeholders and their roles, aspirations, powers and on how they converge and conflict.*

### Philosophical critique: values and pluralism

We have argued that the meaning of concepts depends on their context of use, and that this in turn importantly depends on the purposes of people. Now we want to suggest that stakeholders and their interests are also contextual and relational, thus requiring a form of analysis that accounts for such pluralism. Schleidgen and colleagues at the beginning of their paper suggest a view of medicine as a practice centred on one fundamental goal (meeting patients’ needs) and that other interests, especially economic ones, can only be disturbances to be kept out. We want to show that this is a simplistic and false picture and that stakeholders have goals and purposes (beyond the health of patients) that are perfectly legitimate and need to be recognised. Furthermore, the interests of patients and stakeholders cannot be determined in the abstract: they need to be worked out in the appropriate contexts.

It should be noticed that not all medical needs are equally urgent and serious, especially when health needs are supply-induced (e.g. by pharmaceutical industry [73, 74]). Thinking that patients’ interests always and necessarily override any other interests is both unrealistic and morally unwarranted. Arguably some non-vital patients’ interests have no claim on doctors’ time or on taxpayers’ money. So all we need to say is that patients’ serious interests have *prima facie* priority, but can occasionally be overridden by other legitimate stakeholders’ interests, *when a convincing case for these latter can be made*.

Moreover, to claim that one particular value or interest is central and articulates the goal and the *ethos* of a particular activity (e.g. medicine) does not entail that any other value and interest is always and necessarily subordinated and overridden, let alone irrelevant. On the contrary, other values and interests constitute the framework of constraints within which any activity is *itself* sustainable, permissible and valuable. So for instance, the extremely long working hours of junior doctors (or residents, as they are called in the USA) may be criticised because they jeopardise patients’ safety [75], but also because they are exploitative.^13^ However, the view that stakeholders’ interests have no place in medicine would disallow the latter reason of complaint. We can make the point in loftier Kantian terms: stakeholders should not be treated simply as means (for promoting health), but also always like ends in themselves, i.e. like people with their own aims to pursue. But to have goals implies being interested in the means to achieve them, and when resources are not unlimited this leads to concerns about their distribution, i.e. to economic issues.

While we share Schleidgen and colleagues’ concern that the economic interests of, say, the pharmaceutical and biotech industries may orient PM too much in view of their own profit, we believe that bluntly condemning (direct or indirect) economic interests gives too simplistic and moralistic a picture. We rather need to make an honest appraisal of economic interests and acknowledge that, within proper limits, they are legitimate. Surely it is not always wrong for taxpayers to be concerned about escalating health costs, for patients to worry about out-of-pocket payments or loss of working days and income, for policy-makers to wonder about the sustainability and opportunity costs of endorsing a new health technology, for industries to aim for a profit.

Furthermore, as we have anticipated, stakeholders’ goals and preferences too should be seen as context-dependent: they emerge in real situations as responses to problems, challenges and conflicts. Doctors are doctors when lobbying against health care cuts, but they are cardiologists, oncologists and pediatricians when making claims about how the health budget should be allocated. Patients are sick people, but they are also tax-payers and perhaps parents concerned about the future sustainability of the healthcare system. It is in response to concrete difficulties, hard choices and rival claims that groups of stakeholders get formed, are defined and articulate their aspirations. Since stakeholders’ interests are not necessarily illegitimate and they are not given and fixed, we can appreciate that the plurality of visions of PM is not only an unfortunate source of confusion, but also the result of a variety of concerns and aspirations being articulated.

If we have a closer look at the implications of acknowledging the context-dependence of stakeholders’ interests, we should realise that these contexts are socially constructed and that this means that they are framed by other stakeholders who have set the agenda and the discursive space. Contexts come with biases: to reset them in neutral terms is rarely, if ever, possible. This is why seeing PM as an open negotiable concept not only reflects current reality better, but may also be useful and enrich the ethical reflection around PM: accepting a plurality of visions allows for a variety of *fora* where a multiplicity of views is voiced, so that gatekeeping and framing is not monopolised by any one group or coalition. The openness and negotiability of “PM” may help to prevent hegemonic control over it and capture of the concept by some stakeholders (cf. the concept of disciplinary capture [76, 77]). Our claim is that the current plurality of visions of PM may not only be an annoyance and an obstacle—an unfortunate source of confusion—but may also be the result of a variety of concerns and aspirations being articulated by different stakeholders in different contexts. If this is the case, imposing a more consistent view may not be a desirable solution: we suggest instead that we accept PM as an open negotiable concept. This would be no panacea, but rather a coping strategy that is still preferable to a very invasive therapy.

## Conclusions

Our task has been largely critical, because we believe that in the literature about PM there are too many attempts to bring order and clarity without really understanding the deep roots of the disputes and that any semantic claim has deep normative implications. For instance if, against all odds, Schleidgen and colleagues’ attempt were successful, it would decide against those who argue for a not merely molecular view of the personalisation of medicine without even having given a fair hearing to their arguments. Schleidgen and colleagues have pursued the task of “sharpening a vague term” with the goal of preventing stakeholders from appropriating it. However they have ended up embracing the point of view of some stakeholders without realizing that the appropriation of the concept by those very stakeholders is itself a cause of contention. Furthermore, and ironically enough, in spite of their (overstated) claim that patients’ benefits override any other stakeholders’ interests, they have completely excluded the voice of patients from the construction of their definition.

We suggest that in dealing with personalised medicine it is wise to turn on its head Marx’s famous statement that it is time for philosophers to move from interpreting the world to changing it. So instead of attempting to impose a particular view of PM it would be wiser to be more modest and more open. We should acknowledge the fuzziness, the differences as well as the similarities between many views and visions of PM voiced in different circumstances. A thorough and (as far as possible) unbiased description of this cluster or family of related, overlapping and sometimes contradictory understandings of PM (and its relations to neighbouring concepts like *precision medicine*, *stratified medicine*, and *patient-centered medicine*) seems the appropriate starting point for any philosophical clarification of the concept of PM. Any attempt to go beyond a description of the situation and an interpretation of its drivers becomes a normative attempt and as such needs to engage explicitly with competing normative claims. When language and politics are intermingled—as is the case with PM—there is no such thing as purely logical tidying up. What is in dispute are not merely terminological disagreements, but are different values, ideals and allocations of powers and resources. The question “What is PM?” cannot be separated from questions like “Who contributes what to PM?”, “Who is setting the agenda?”, “Who has been excluded?”, “Who gets what?”. None of these questions is settled yet, and so it seems to us that rather than trying to pin down an elusive concept, it would be worth mapping stakeholders’ aspirations and interests and try to contribute to make the negotiations about the future of PM as fair and transparent as possible.

## Abbreviations

PM, personalised medicine; RCT, randomised controlled trial; SLR, systematic literature review.

## Footnotes

1 The European Commission recently endowed the call for research on Personalised Medicine with € 659 million [78], and claims to have already committed more than € 1 billion since 2007 [79]. In the US president Obama in the 2015 State of the Union launched the Precision Medicine Initiative (precision medicine is part of Personalised Medicine as we use the term in this paper) and then endowed it with $ 215 million [80–82].

2 It should be noted that another driver of PM is the development of self-tracking devices that allow individuals to collect data and information that could be used to monitor and promote health [83–88]. This trend is usually subsumed under the concept of the Quantified Self (or self-tracking) and linked to the concept of PM especially in the literature aiming at popularising the concept [14]. While it is likely that we are going towards an integration of information coming from molecular biology and information coming from personal wearable or implanted devices, in the present paper we focus on molecular biology and leave aside self-tracking technologies.

3 On April 4 2016 the article boosted 50 citations according to Google scholar, 16 citing articles according to Web of Science, 7792 accesses according to *BMC Medical Ethics*, 24 citations according to the ranking of bioethical articles produced by Georgetown University Research Library, where the paper ranked 19th for the year 2013 (thus looking like the most influential bioethical publication on the topic of defining PM), and it has been included in a recently published anthology on *The Ethics of Personalised Medicine* [89].

4 A reviewer remarked that the word “utilizing” in the definition may suggest that they include translation, implementation etc. The reason why we have not followed this charitable reading is that Schleidgen and colleagues understand the possibilities and limitations of PM only in techno-scientific terms and see PM as just an add-on to current practice, thus seeming to rule out the need for structural changes and downplay the importance of organisational and financial adjustments demanded by PM.

5 Our objection is not against taking a stance. But when taking sides in a debate, one should do so not pretending to be merely doing logical tidying up, but should fully engage with the normative claims in the debate.

6 Clearly this is an oversimplification. In certain cases, for instance when external validity is more important than internal validity, other design studies may be preferable to RCTs. However, here our point is simply that if the sources reviewed lack robustness, the final result cannot claim much strength.

7 The same kind of sensitivity should be used in understanding what kind of academic papers one is reviewing. Reviews, editorials and commentaries are all academic subgenres, but differ radically from research articles, and the criteria to judge how reliable and robust they are vary accordingly. For instance experimental results have different weight if they report an early observation of a phenomenon or whether they confirm very compellingly earlier more tentative studies. An editorial or a commentary has to be read differently when it is about a scientific issue that is controversial or about one over which there is a settled consensus. The role of different genres of scientific writing in PM is stressed by Adam Hedgecoe [54].

8 Here again the problem is that they have not distinguished between concepts and names. It is indeed possible that PM is sometimes used to refer to an old concept and sometimes to a new concept [4, 90], and it is certainly possible that there are sensible criteria for naming that suggest to use PM for the old concept and a new name for the new concept. If they wanted to give a fair refutation of the view that PM is nothing new, they should have shown that PM (broadly defined to accommodate common current uses) cannot be used to identify pre-existing medical practices because such practices are already covered by an existing concept different from PM. If this were the case this proposed use of PM for old medical practices would be unnecessary, redundant and confusing. But they have not shown that. It is also worth noting that some proponents of PM (e.g. [69]) have explicitly presented it as the scientific culmination of the time-honoured medical habit of adapting knowledge to individual cases. For a discussion of this dialectical tension between PM as revolutionary and as consistent with the medical tradition see [11, 14, 15].

9 For the articulation of a broader and historically rooted view of medicine that includes not only curing and preventing but also relieving and comforting see [91].

10 It is worth remembering that the genomic understanding of PM first appeared in 1997 in an article by Andrew Marshall [92] who used the concept in the plural (“personalized medicines”) and in his next publication dropped it in favour of “pharmacogenomics”. The paper that really kicked-off the new understanding of PM was Langreth and Waldholz’s “New Era of Personalized Medicine” which appeared in 1999 in the *Wall Street Journal* and shortly after was reprinted by *The Oncologist* [93]. This paper reports the strategies and alliances that pharmaceutical industries had launched on in pursuing new opportunities in pharmacogenomics (incidentally the opportunities for pharmaceutical industry were given as much emphasis, if not more, as benefits for patients already in Marshall’s 1997 paper). This should remind us that stakeholders’ interests have contributed from the start to shape the visions of PM and that many researchers pioneering PM were working for pharmaceutical or biotech companies, making it hard to separate science from industry (cf. [58]).

11 Though one may assume the authors think everyone *ought* to accept their definition, they have no illusions that in fact they will; however they do claim that their definition ought to be regarded as *acceptable* by the majority ([12]: p. 10).

12 For instance Richard Tutton noted (in 2012, before Schleidgen and colleagues published their paper) that “there have been different historical forms of ‘personalization’ over time” ([11]: p. 1726) and that before the rise of genomic medicine, clinicians had used it to describe a way of practicing medicine “endangered by continued technologization and reductionism of biomedicine” ([11]: p. 1721). A recent article published in such a prestigious journal as *The Lancet* [94] shows that this understanding of PM has not disappeared. Hedgecoe and Martin [59] have shown that even within that subset of PM represented by pharmacogenetics, alternative visions of its mission and scope exist and that these latter are affected by the coalitions that support them and can be modified when this latter change. “For example, as new groups of actors join the emerging network, they may favour particular options over others and shape the future direction of research” ([59]: p. 330). These examples suggest that PM is an evolving concept, still fluid and whose meaning is not, and cannot be, fixed yet.

13 It has been noted that junior doctors have fewer protections than workers in sweatshops [95] and prisoners of war [96].

## References

[1] Ruaño G (2004). Quo vadis personalized medicine?. Pers Med.

[2] Pray L (2008). Personalized medicine: Hope or hype. Nat Educ.

[3] Ginsburg GS, Willard HF (2009). Genomic and personalized medicine: foundations and applications. Transl Res.

[4] Steele FR (2009). Personalized medicine: something old, something new. Pers Med.

[5] Laurence J (2009). Getting personal: the promises and pitfalls of personalized medicine. Transl Res.

[6] Samani NJ, Tomaszewski M, Schunkert H (2010). The personal genome--the future of personalised medicine?. Lancet.

[7] Salari K, Watkins H, Ashley EA (2012). Personalized medicine: hope or hype?. Eur Heart J.

[8] Doz F, Marvanne P, Fagot-Largeault A (2013). The person in personalised medicine. Eur J Cancer.

[9] Nicholls SG, Wilson BJ, Castle D, Etchegary H, Carroll JC (2014). Personalized medicine and genome-based treatments: why personalized medicine ≠ individualized treatments. Clin. Ethics.

[10] Jørgensen JT (2009). New era of personalized medicine: a 10-year anniversary. Oncologist.

[11] Tutton R (2012). Personalizing medicine: Futures, present and past. Soc Sci Med.

[12] Schleidgen S, Klingler C, Bertram T, Rogowski WH, Marckmann G (2013). What is personalized medicine: sharpening a vague term based on a systematic literature review. BMC Med Ethics.

[13] Pokorska-Bocci A, Stewart A, Sagoo GS, Hall A, Kroese M, Burton H (2014). ‘Personalized medicine’: what’s in a name?. Pers Med.

[14] Michl S, Fischer T, Langanke M, Marschall R, Michl S (2015). Inventing traditions, raising expectations. Recent debates on ‘Personalized Medicine’. Individualized Medicine; Ethical, economical and historic perspectives.

[15] Billaud M, Guchet X (2015). L’invention de la médecine personnalisée. Méd/Sci.

[16] Cribb A, Owens J (2010). Whatever suits you: unpicking personalization for the NHS. J Eval Clin Pract.

[17] Simmons LA, Dinan MA, Robinson TJ, Snyderman R (2012). Personalized medicine is more than genomic medicine: confusion over terminology impedes progress towards personalized healthcare. Pers Med.

[18] Redekop WK, Mladsi D (2013). The faces of personalized medicine: a framework for understanding its meaning and scope. Value Health.

[19] Cherny NI, de Vries EG, Emanuel L, Fallowfield L, Francis PA, Gabizon A, et al. Words matter: distinguishing “personalized medicine” and “biologically personalized therapeutics”. JNCI J Natl Cancer Inst. 2014;106–12. doi:10.1093/jnci/dju321. 10.1093/jnci/dju321 PMC4568994 25293984

[20] Langanke M, Lieb W, Erdmann P, Dörr M, Fischer T, Kroemer HK, Fischer T, Langanke M, Marschall R, Michl S (2015). The meaning of “Individualized Medicine”: A terminological adjustment of a perplexing term. Individualized medicine; ethical, economical and historic perspectives.

[21] Harvey A, Brand A, Holgate ST, Kristiansen LV, Lehrach H, Palotie A, Prainsack B (2012). The future of technologies for personalised medicine. New Biotechnol.

[22] Khoury MJ, Gwinn M, Yoon PW, Dowling N, Moore CA, Bradley L (2007). The continuum of translation research in genomic medicine: how can we accelerate the appropriate integration of human genome discoveries into health care and disease prevention?. Genet Med.

[23] Downing GJ (2009). Key aspects of health system change on the path to personalized medicine. Transl Res.

[24] Snyderman R, Dinan MA (2010). Improving health by taking it personally. Jama.

[25] Mirnezami R, Nicholson J, Darzi A (2012). Preparing for precision medicine. N Engl J Med.

[26] Issa AM (2015). 10 years of personalizing medicine: how the incorporation of genomic information is changing practice and policy. Pers Med.

[27] Leyens L, Horgan D, Lal JA, Steinhausen K, Satyamoorthy K, Brand A (2014). Working towards personalization in medicine: main obstacles to reaching this vision from today’s perspective. Pers Med.

[28] Snyderman R, Williams RS (2003). Prospective medicine: the next health care transformation. Acad Med.

[29] Gorini A, Pravettoni G (2011). P5 medicine: a plus for a personalized approach to oncology. Nat Rev Clin Oncol.

[30] Pravettoni G, Gorini A (2011). A P5 cancer medicine approach: why personalized medicine cannot ignore psychology. J Eval Clin Pract.

[31] Burnette R, Simmons LA, Snyderman R (2012). Personalized health care as a pathway for the adoption of genomic medicine. J Personalized Med.

[32] Cornetta K, Brown CG (2013). Perspective: balancing personalized medicine and personalized care. Acad Med.

[33] Horwitz RI, Cullen MR, Abell J, Christian JB (2013). (De)personalized medicine. Science.

[34] Heusser P, Vollmann J, Sandow V, Wäscher S, Schildmann J (2015). Towards integration of personalised and ‘person-centred’ medicine: The concept of ‘integrative and personalised health care’. The Ethics of Personalised Medicine.

[35] D’Abramo F, Goerling U, Guastadisegni C (2016). Targeted drugs and Psycho-oncological intervention for breast cancer patients. J. Negat. Results Biomed..

[36] Abettan C (2016). Between hype and hope: What is really at stake with personalized medicine?. Med Health Care Philos.

[37] Burke W, Psaty BM (2007). Personalized medicine in the era of genomics. JAMA.

[38] Burke W, Trinidad SB, Press NA (2014). Essential elements of personalized medicine. Urol Oncol.

[39] McFarland DC, Hamilton JG, Fox R, Holland J (2014). Putting the “Person” in personalized cancer medicine: A systematic review of psychological aspects of targeted therapy. Personalized Med Oncol.

[40] Tutton R (2014). Genomics and the reimagining of personalized medicine.

[41] Bos C, Walhout B, Peine A, van Lente H (2014). Steering with big words: articulating ideographs in research programs. J Responsible Innov.

[42] The Cochrane Collaboration. What is Cochrane evidence and how can it help you? http://www.cochrane.org/what-is-cochrane-evidence. Accessed 4 Apr 2016.

[43] The Campbell Collaboration. What is a systematic review? http://www.campbellcollaboration.org/what_is_a_systematic_review/index.ph. Accessed 4 Apr 2016.

[44] Slavin RE (1995). Best evidence synthesis: an intelligent alternative to meta-analysis. J Clin Epidemiol.

[45] Torgerson C (2003). Systematic reviews.

[46] Davis JC, Furstenthal L, Desai AA, Norris T, Sutaria S, Fleming E, Ma P (2009). The microeconomics of personalized medicine: today’s challenge and tomorrow’s promise. Nat Rev Drug Discov.

[47] Sprangers MA, Hall P, Morisky DE, Narrow WE, Dapueto J (2013). Using patient-reported measurement to pave the path towards personalized medicine. Qual Life Res.

[48] Issa AM (2007). Personalized medicine and the practice of medicine in the 21st century. McGill Journal of Medicine.

[49] Gibson WM (1971). Can personalized medicine survive?. Can Fam Physician.

[50] Osborn LA. Psychiatry and medicine: An introduction to personalized medicine. New York: McGraw-Hill Book Company; 1952.

[51] Arnold RM, Forrow L (1990). Rewarding medicine: Good doctors and good behavior. Ann Intern Med.

[52] Robinson GC (1939). The patient as a person: A study of the social aspects of illness.

[53] Ryle JA (1943). Social medicine: its meaning and its scope. Br Med J.

[54] Boniolo G, Sanchini V (eds). Ethical counselling and medical decision-making in the era of personalised medicine: A practice-oriented guide. (s.p.): Springer; 2016.

[55] Gronowicz G (2016). Personalized medicine: Promises and pitfalls.

[56] Katsnelson A (2013). Momentum grows to make ‘personalized’ medicine more ‘precise’. Nat Med.

[57] Munthe C. Personalised, individualised or precision medicine: Three unaddressed socio-economic hurdles. Philos Comment. 2016;6. http://philosophicalcomment.blogspot.no/2016/03/personalised-individualised-or.html.

[58] Hedgecoe A (2004). The politics of personalised medicine: Pharmacogenetics in the clinic.

[59] Hedgecoe A, Martin P (2003). The drugs don’t work expectations and the shaping of pharmacogenetics. Soc Stud Sci.

[60] Hedgecoe A (2007). Education, ethics and knowledge deficits in clinical pharmacogenetics. Pharmacogenomics.

[61] Petersen A (2009). The ethics of expectations. Monash Bioeth Rev.

[62] Michl S, Fischer T, Langanke M, Marschall R, Michl S (2015). The epistemics of ‘Personalized Medicine’. Rebranding pharmacogenetics. Individualized medicine; ethical, economical and historic perspectives.

[63] Gray SW, Hicks-Courant K, Lathan CS, Garraway L, Park ER, Weeks JC (2012). Attutudes of patients with cancer about personalized medicine and somatic genetic testing. J Oncol Pract.

[64] Wittgenstein L (2009). Philosophical investigations.

[65] Berne E (1967). Games people play:The psychology of human relationships.

[66] Williamson T (1994). Vagueness.

[67] Levi DS (1996). The unbearable vagueness of being. South J Philos.

[68] Hanfling O (2003). Philosophy and ordinary language: The bent and genius of our tongue.

[69] Roses AD (2000). Pharmacogenetics and the practice of medicine. Nature.

[70] President’s Council of Advisors on Science and Technology. Priorities for personalized medicine. Washington DC; 2008 Sep. https://www.whitehouse.gov/files/documents/ostp/PCAST/pcast_report_v2.pdf

[71] Trusheim MR, Berndt ER, Douglas FL (2007). Stratified medicine: strategic and economic implications of combining drugs and clinical biomarkers. Nat Rev Drug Discov.

[72] Rawls J (1993). Political liberalism.

[73] Moynihan R, Cassels A (2005). Selling sickness: How the world’s biggest pharmaceutical companies are turning us all into patients.

[74] Dumit J (2012). Drugs for life: How pharmaceutical companies define our health.

[75] Temple J (2014). Resident duty hours around the globe: where are we now?. BMC Med Educ.

[76] Gilson L, Hanson K, Sheikh K, Agyepong IA, Ssengooba F, Bennett S (2011). Building the field of health policy and systems research: social science matters. PLoS Med.

[77] Brister E (2016). Disciplinary capture and epistemological obstacles to interdisciplinary research: lessons from central African conservation disputes. Stud Hist Philos Sci Part C.

[78] European Commission. Press release database. http://europa.eu/rapid/press-release_MEMO-15-5832_en.htm (2015 Oct 13). Accessed 3 Apr 2016.

[79] European Commission. Directorate general for health and food. http://ec.europa.eu/health/human-use/personalised-medicine/index_en.htm. Accessed 3 Apr 2016.

[80] The white house. Office of the press secretary. (https://www.whitehouse.gov/the-press-office/2015/01/30/fact-sheet-president-obama-s-precision-medicine-initiative (2015 Jan 30). Accessed 3 Apr 2016.

[81] National Institute of Health. Precision medicine initiative cohort program. Bethesda Ma. https://www.nih.gov/precision-medicine-initiative-cohort-program. Accessed 3 Apr 2016.

[82] Reardon S (2015). Obama to seek $ 215 million for precision medicine plan. Nat New.

[83] Swan M (2012). Crowdsourced health research studies: an important emerging complement to clinical trials in the public health research ecosystem. J Med Internet Res.

[84] Gujarathi R, Costa FF (2014). The impact of online networks and big data in life sciences. Soc Netw.

[85] Dudley JT, Listgarten JE, Stegle OL, Brenner SE, Parts LE, Altman RB (2015). Personalized medicine: from genotypes, molecular phenotypes and the quantified self, towards improved medicine. Pacific Symposium in Biocomputing 2015.

[86] Salamati F, Pasek ZJ (2014). Personal wellness: complex and elusive product and distributed self-services. Procedia CIRP.

[87] Lupton D (2013). Quantifying the body: monitoring and measuring health in the age of mHealth technologies. Crit Pub Health.

[88] Barr AL. Google’s new moonshot project: the human body. Wall Street J. 2014;27.

[89] Vollmann J, Sandow V, Wäscher S, Schildmann J (2015). The ethics of personalised medicine.

[90] Guchet X (2014). Le patient «actionnable» de la médecine personnalisée. Socio-Anthropologie.

[91] Miles A (2009). On a medicine of the whole person: away from scientistic reductionism and towards the embrace of the complex in clinical practice*. J Eval Clin Pract.

[92] Marshall A (1997). Laying the foundations for personalized medicines. Nat Biotechnol.

[93] Langreth R, Waldholz M (1999). New era of personalized medicine targeting drugs for each unique genetic profile. Oncologist.

[94] Patel V (2015). The art of medicine. Rethinking personalised medicine. Lancet.

[95] Anonymous. Medical residents are abused more than Chinese factory workers. KevinMD.com. 2012 Apr 23; http://www.kevinmd.com/blog/2012/04/medical-residents-abused-chinese-factory-workers.html (accessed 23/03/2016).

[96] Moreton A (2014). The acrimonious road to the 48 hour week. BMJ Careers.

